# Role of Cardiac Imaging in the Diagnosis of Immune Checkpoints Inhibitors Related Myocarditis

**DOI:** 10.3389/fonc.2021.640985

**Published:** 2021-05-13

**Authors:** Stéphane Ederhy, Joe-Elie Salem, Laurent Dercle, Abrar Saqif Hasan, Marion Chauvet-Droit, Pascal Nhan, Samy Ammari, Bruno Pinna, Alban Redheuil, Samia Boussouar, Stephane Champiat, Laurie Soulat-Dufour, Ariel Cohen

**Affiliations:** ^1^ Department of Cardiology, Sorbonne Université, AP-HP, Saint-Antoine Hospital, Paris, France; ^2^ UNICO-GRECO APHP.Sorbonne Cardio-Oncology Program, Sorbonne Université, Paris, France; ^3^ Sorbonne Université, INSERM CIC-1901, AP-HP.Sorbonne, Department of Pharmacology, Pitié-Salpêtrière Hospital, Paris, France; ^4^ Unité INSERM UMRS-ICAN 1166, Unité de recherche sur les maladies cardiovasculaires, du métabolisme et de la nutrition, Sorbonne Universités, Paris, France; ^5^ Division of Medicine and Pharmacology, Cardio-oncology program, Vanderbilt University Medical Center, Nashville, TN, United States; ^6^ Department of Radiology, New York Presbyterian, Columbia University Irving Medical Center, New York, NY, United States; ^7^ Department of Internal Medicine, Montefiore/Albert Einstein College of Medicine, Bronx, NY, United States; ^8^ Radiology Department, Gustave Roussy Cancer Campus, Villejuif, France; ^9^ BIOMAPS, UMR1281, INSERM.CEA.CNRS, Université Paris-Saclay, Paris, France; ^10^ LIB Biomedical Imaging Laboratory INSERM, CNRS, ICT Cardiothoracic Imaging Unit & Radiology Department, ICAN Institute of Cardiometabolism and Nutrition, Pitié-Salpêtrière Hospital (AP-HP), Sorbonne Université, Paris, France; ^11^ Drug Development Department (DITEP), Institut Gustave Roussy, Villejuif, France

**Keywords:** myocarditis, immune checkpoint inhibitor, cancer, cardiac magnetic resonance imaging, cardiotoxicity

## Abstract

Immune checkpoint inhibitors (ICI) have constituted a paradigm shift in the management of patients with cancer. Their administration is associated with a new spectrum of immune-related toxicities that can affect any organ. In patients treated with ICI, cardiovascular toxicities, particularly myocarditis, occur with a low incidence (<1%) but with a high fatality rate (30−50%). ICI-related myocarditis has been attributed to an immune infiltration, comprising of T-cells that are positive for CD3+, CD4+, CD8+, and macrophages that are positive for CD68. The diagnosis remains challenging and is made based on clinical syndrome, an electrocardiogram (ECG), biomarker data, and imaging criteria. In most clinical scenarios, endomyocardial biopsy plays a pivotal role in diagnosis, while cardiac magnetic resonance imaging (cMRI) has limitations that should be acknowledged. In this review, we discuss the role of medical imaging in optimizing the management of ICI related myocarditis, including diagnosis, prognostication, and treatment decisions.

## Introduction

Immune checkpoint inhibitors (ICIs) have modified the management of patients with cancer and have improved their prognosis and survival for many tumor types including melanoma, lymphoma, kidney, and lung malignancies (1). From a mechanistic point of view, ICIs are monoclonal antibodies that antagonize the pathways for programmed cell death receptor 1 (PD‐1), programmed cell death ligand 1 (PD‐L1), cytotoxic T‐lymphocyte–associated protein 4 (CTLA‐4) and could activate the immune system against cancer cells (2–4).

ICI administration can lead to immune-related adverse events (irAE) that can potentially affect all organs (5, 6). Among them, ICI related myocarditis is a rare adverse event that has an estimated incidence between 0.09 to 1.14% (7, 8). The incidence of myocarditis is higher in patients treated with ICI administered in combinations (e.g., anti-PD-1 and anti-CTLA-4) (0.27%) than in monotherapy with ICI (0.09%) (5). An early onset characterizes ICI related myocarditis, generally occurring within the first three injections (9).

ICI-related myocarditis is associated with poor outcomes, since the fatality rate has been reported to range between 30 to 50% (3, 9). Hence, early diagnosis and management is crucial. ICI-related myocarditis is frequently associated with other irAE, such as myositis (in 25.4%), myasthenia-like syndromes (ptosis, diplopia, respiratory failure; in 10.7%), and hepatitis (in 10.7%) (9). There is a growing body of evidence indicating that ICI-related myocarditis should be considered as a new entity and etiology of acute myocarditis, that differs from other etiologies by several aspects, including clinical presentation, ECG, and cardiac magnetic resonance imaging (cMRI) features (10). In this review, we discuss medical imaging’s role in optimizing the management of ICI related myocarditis, including diagnosis, prognostication, treatment decision, and follow-up.

## Diagnostic Criteria for ICI-Related Myocarditis

According to the ESC 2013 position statement, the diagnosis of acute myocarditis relies on a combination of a suggestive clinical presentation, first-line tests such as ECG, biomarkers including inflammatory markers, viral antibodies, serum cardiac antibodies, and cardiac biomarkers (Troponin I or T, BNP, or NT pro BNP), transthoracic echocardiography (TTE), and cMRI. It also includes second-line tests, represented by coronary angiography (CA), to exclude coronary artery disease and endomyocardial biopsy (EMB) (11). This diagnostic workup could be applied to document ICI-related myocarditis, but it should also be adapted to the specific context of cancer patients treated with ICI, as specified in a recent statement (10).

## Clinical Presentation

The clinical presentation in a patient admitted for acute myocarditis (AM) includes a wide range of symptoms. On one hand, some patients are pauci symptomatic on presentation with chest pain and/or palpitations. On the other hand, there are more severe scenarios with cardiogenic shock (11). Discussing out of the context of cancer patients treated with ICI, five possible clinical scenarios could be identified in acute myocarditis patients. They include acute coronary like syndrome, new onset or worsening heart failure, chronic heart failure, and life-threatening conditions including arrhythmia, sudden cardiac arrest, and cardiogenic shock with impaired left ventricular systolic dysfunction (11).

In cancer patients treated with ICI, the clinical presentation of ICI-related myocarditis has recently been described by Pradhan et al. in a review gathering data from 88 published cases. They found that the most commonly reported symptoms in ICI-related myocarditis were dyspnea (49%), weakness (25%), chest pain (17%), syncope (9%), fever (6%), and cough (4%) (12). Multiple recent publications show that ICI-related myocarditis could have several other presentations. There is a growing body of evidence suggesting that patients presenting with an irAE could have a final diagnosis of AM without any evidence of clinical cardiac manifestations and that some patients could have isolated troponin rise and/or ECG modifications (13).

## ECG Findings

ECG is generally abnormal, but it should be noticed that ECG features are neither specific nor sensitive enough for the diagnosis of AM. Main ECG findings that could be encountered in patients with acute myocarditis are the following: 1st, 2^nd^, and 3rd degree atrioventricular block, bundle branch block, ST/T wave modifications, ST elevation (generally concave and non-mirror), non-ST elevation, T wave inversion, sinus arrest, ventricular tachycardia or fibrillation, asystole, atrial fibrillation, intraventricular conduction delay, abnormal Q waves, premature beats, and SVT (14). However, a normal ECG does not rule out the diagnosis of acute myocarditis. In a study of 77 patients with acute myocarditis, ECGs were normal in 32% of the patients, ST elevation was found in 57%, inverted T wave in 9%, and left bundle branch block in 3% (15). In a similar fashion, for a study of 65 patients with biopsy-proven myocarditis, ST-abnormalities were detected in 69% of the patients, bundle-branch-block in 26%, Q-waves in 8%, atrial fibrillation was present in 6%, and AV-Block in 3% of the patients (16).

In cancer patients with ICI-related myocarditis, Pradhan showed that 91% of ECGs were found to be abnormal and that there was a broad spectrum of abnormal findings. ST-elevation was reported in 32% and ST-depression in 4% cases. Various degrees of heart block were found in 51%, with complete AV block involving 66% of them. Ventricular tachycardia or fibrillation were noted in 35% (12).

## Transthoracic Echocardiography

Transthoracic echocardiography (TTE) represents the first line of imaging when AM is clinically suspected. It has the advantage of being non-invasive, non-ionizing, versatile, and available at bedside. TTE provides information on cardiac geometry, morphology, and function. TTE findings suggestive of acute myocarditis encompass segmental wall motion abnormalities, increased LV wall thickness, global hypokinesia, particularly in fulminant myocarditis, and pericardial effusion. The current role of speckle tracking imaging is not clearly established in this context. A normal TTE does not rule out the diagnosis of acute myocarditis (17).

Out of the context of immunotherapy, in a case series of 41 patients with histologically proven myocarditis, left ventricular dysfunction was noticed in 69%, right ventricular dysfunction in 23%, wall motion abnormality in 64%, left ventricular “pseudo hypertrophy” in 20%, and ventricular thrombi in 15% (15). Felker described the TTE features in the vast majority of patients admitted for fulminant myocarditis, as showing an increased septal thickness and normal LV dimension. In contrast, those with non-fulminant myocarditis had an increased diastolic dimension with a normal septal thickness (18).

In cancer patients with ICI-related myocarditis, there are scarce data on TTE findings. In a recent review of the literature describing TTE findings in 53 myocarditis cases, 23% of TTE were classified as normal, and 32.5% of TTE examinations reported a normal LVEF (12).

## Cardiac Magnetic Resonance and ICI-Related Myocarditis

cMRI as TTE allows to define cardiac geometry, morphology, function and add important information by allowing myocardial tissue characterization, particularly in the context of inflammation related to myocarditis. The combination of markers of edema and inflammation increases the probability of AM. According to the updated 2018 Lake Louise criteria, at least one T2 based criterion (a regional or global increase of myocardial T2 relaxation time or increased signal intensity in T2-weighted CMR images) with at least one T1 based criterion (increase myocardial T1, extracellular volume, or late gadolinium enhancement) should be analyzed and combined to improve the diagnostic accuracy of cMRI for the diagnosis of AM (19, 20).

In cancer patients with ICI-related myocarditis, the most extensive description of cMRI findings was made by Zhang (21). LGE was found in 48% of all the cases, was predominantly distributed at the anteroseptal, inferoseptal, inferior, and inferolateral segments. Myocardial edema, as assessed with T2 weighted STIR was found in only 28% of cases. Forty-three patients had neither elevated T2 nor LGE. The predominant LGE pattern was subendocardial/transmural in 6.1%, subepicardial in 26.5%, mid-myocardial in 49%, and diffuse in 18.4%. Native T1 value was comparable (18). However, it should be noticed that a normal cMRI with a normal T2, T1 values and no LGE does not rule out ICI myocarditis proven with BEM (21, 22).

## Endomyocardial Biopsy and Acute Myocarditis

Acute myocarditis, according to the 2013 ESC position statement, is defined as an inflammatory disease of the myocardium diagnosed by histological evidence of inflammatory infiltrates defined as ≥14 leucocytes/mm², including up to 4 monocytes/mm² with the presence of CD 3 positive T-lymphocyte ≥7 cells/mm², within the myocardium associated with myocyte degeneration and necrosis of non-ischemic origin (11).

Endomyocardial biopsy is necessary to achieve a diagnosis of certainty and identify its cause. Causes of myocarditis include infectious myocarditis (bacterial, spirochetal, viral), immune-mediated myocarditis (allergens, alloantigens, autoantigens), and toxic myocarditis (drugs, heavy metals, hormones, physical agents, miscellaneous) (11). It is also essential to bear in mind that cancer patients developing myocarditis could have other possible etiologies, including radiotherapy, anthracycline, or other viral infections. (23).

In cancer patients with ICI-related myocarditis, postmortem histopathological analysis of heart and skeletal biopsies in two patients treated with combination therapy (ipilimumab and nivolumab) revealed the myocardium as necrotized and associated with an intense, patchy, lymphocytic infiltrate. The infiltrate comprised T-cells positive for CD3+, CD4+, and CD8+, and macrophages positive for CD68. PD-L1 was expressed on myocytes’ membranous surface, on infiltrating CD8+ T-cells, but was not expressed on skeletal muscle or tumor (7).

Of note, in expert centers, the complication rate of EMB is low and is influenced by operator experience, the volume of procedures and a learning curve. Incidence of peri-procedural complications (perforation, tamponade, embolization) after left ventricular EMB and right ventricular was low and comparable (0.33% for left ventricular EMB and 0.45% for right ventricular EMB) with no death occurring following EMB (24).

## Imaging and Prognosis in ICI-Related Myocarditis

There is scarce data in the context of ICI-related myocarditis on prognostic markers. Transthoracic echocardiography, mainly left ventricular ejection fraction (LVEF), global longitudinal strain (GLS), and abnormal findings on CMR were evaluated as potential prognostic markers in patients with ICI-related myocarditis (25).

In cancer patients with ICI-related myocarditis, Pradhan et al. in a review of 88 cases, published that LVEF was not a predictor of poor outcomes (12). Awadalla et al. showed that MACE’s risk was higher with a lower GLS among patients with either a reduced or preserved left ventricular ejection fraction. After adjustment for ejection fraction, each percent reduction in GLS was associated with a 1.5-fold increase in MACE among patients with a reduced left ventricular ejection fraction (HR 1.5, 95% CI 1.2−1.8) and a 4.4-fold increase with a preserved left ventricular ejection fraction (HR 4.4, 95% CI 2.4−7.8) (25). Regarding CMR parameters, the presence of LGE, LGE pattern, and elevated T2-weighted short TI inversion recovery were not associated with MACE (21).

## Towards a Pragmatic Approach on How to Diagnose ICI-Related Myocarditis

The diagnosis of ICI-related myocarditis relies on combining a clinical syndrome, ECG, troponin measurement, and imaging criteria. Most recent ESMO and ASCO guidelines dealing with the management of ICI-related myocarditis do not describe any diagnostic workup (26, 27). However, recent position statements from the European heart failure association suggest, despite clear evidences, to obtain references values before ICI initiation based on an echocardiogram, an electrocardiogram, and biomarkers measurement (troponin and a natriuretic peptide) (28, 29).

In symptomatic patients, Bonaca intended to describe particularly in the context of immunotherapy, the diagnostic criteria that should be used both in everyday clinical practice and in clinical trials (10).

**Figure 1:**
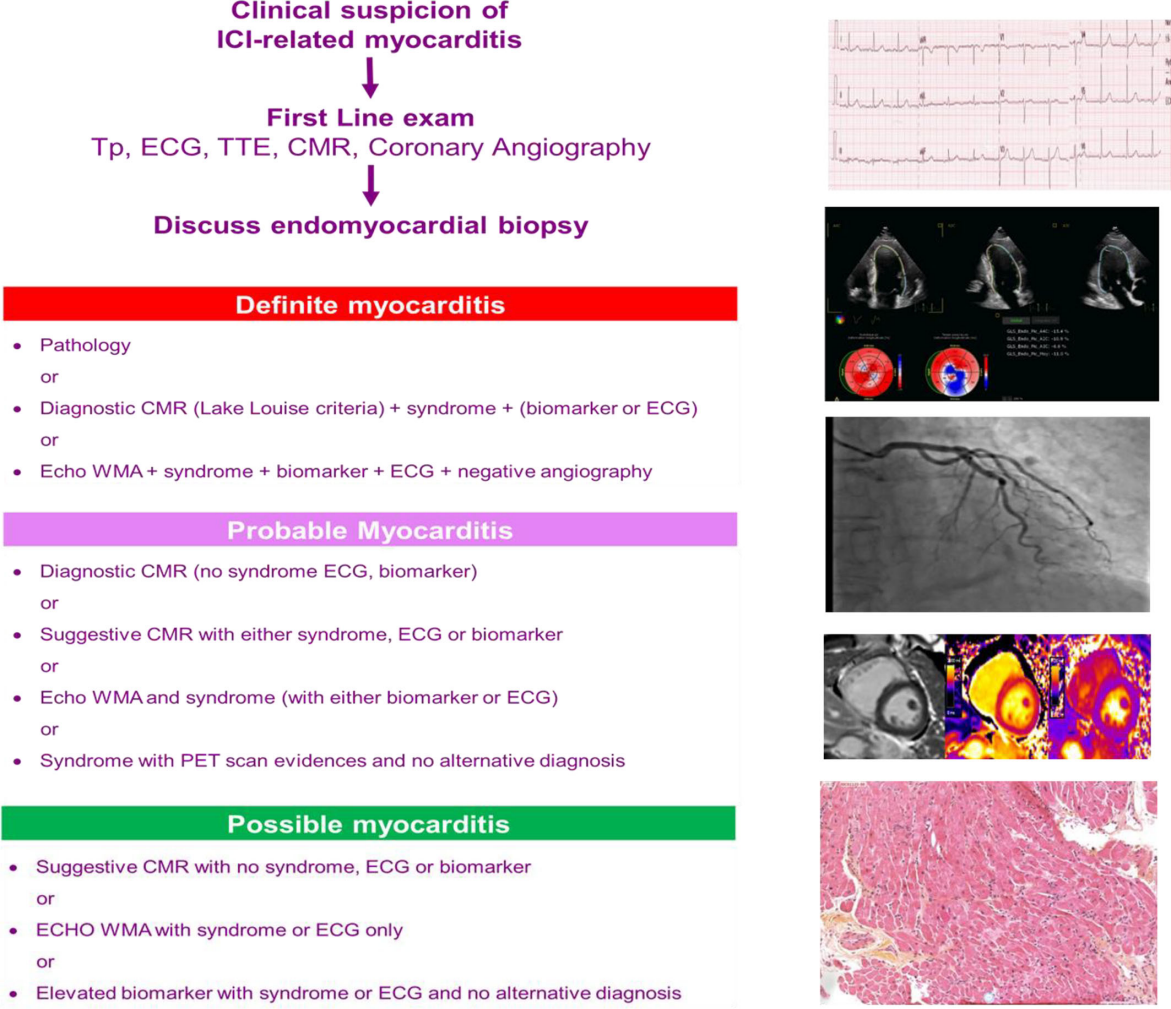
CMR, cardiac magnetic resonance; ECG, electrocardiogram; EMB, endomyocardial biopsy; ICI, immune checkpoint inhibitor; Tn, troponin; TTE, transthoracic echocardiography.

**Figure 1** depicts a diagnostic workup adapted from Bonaca et al. and proposes that when the diagnosis of ICI-related myocarditis is suspected, patients should be evaluated with at least one ECG and a troponin measurement and should be rapidly referred to a cardio-oncology unit that can confirm or exclude the diagnosis of ICI-associated myocarditis (10).

## Conclusions

The incidence of ICI-associated myocarditis is low (below 1%) but could be underestimated since it is not systematically screened. It is critical to diagnose this irAE at an early stage since it is associated with a fatality rate between 30 to 50%. Current strategies usually rely on a suspicion by the patient’s oncologist and a confirmation by a cardiologist or cardio-oncologist based on CMR or endomyocardial biopsies. The diagnosis of ICI-related myocarditis remains challenging and the main objective is to make an early diagnosis since no predictive markers are currently available to identify patients prone to develop ICI related myocarditis.

## Author Contributions

Writing: SE, J-ES, LD, AC. All authors contributed to the article and approved the submitted version.

## Conflict of Interest

The authors declare that the research was conducted in the absence of any commercial or financial relationships that could be construed as a potential conflict of interest.
